# Enzalutamide Plus ADT for the Treatment of mHSPC: Real-World Evidence from a Bulgarian Cohort

**DOI:** 10.3390/cancers18183007

**Published:** 2026-09-16

**Authors:** Manoela Manova, Boryana Ivanova, Antoan Rangelov, Mila Petrova, Assen Dudov, Ivsan Chan, Sevil Ahmed, Victor Nenov, Elitsa Varbanova, Todor Georgiev, Daniel Penchev, Alexandra Savova

**Affiliations:** 1 Department of Organization and Economy of Pharmacy, Faculty of Pharmacy, Medical University, 1000 Sofia, Bulgaria; 2 National Council on Prices and Reimbursement of Medicinal Products, 1431 Sofia, Bulgaria; 3 Faculty of Chemistry and Pharmacy, Sofia University “St. Kliment Ohridski”, 1164 Sofia, Bulgaria; 4 Medical Oncology Clinic, Multiprofile Hospital for Active Treatment (MHAT) Nadezhda, 1330 Sofia, Bulgaria; 5 Medical Oncology Clinic, Acibadem City Clinic Mladost (UMHAT), 1784 Sofia, Bulgaria; 6 Sqilline Health, 1766 Sofia, Bulgaria

**Keywords:** enzalutamide, androgen deprivation therapy, metastatic hormone-sensitive prostate cancer, androgen receptor signaling inhibitor, real-world, ARCHES

## Abstract

**Background/Objectives**: The pivotal ARCHES trial demonstrated the clinical benefit of the second-generation androgen receptor signaling inhibitor enzalutamide in combination with androgen deprivation therapy (ADT) for the treatment of metastatic hormone-sensitive prostate cancer (mHSPC). However, real-world evidence on the efficacy of enzalutamide plus ADT in mHSPC remains very limited. The current work provides real-world data on the efficacy of this combination in a Bulgarian cohort of patients with mHSPC. **Methods**: Patients treated with enzalutamide plus ADT across 48 hospitals between 1 January 2022 and 31 December 2025 were included in our retrospective analysis. Data were extracted from electronic health records, for which large language model (LLM) algorithms were employed, utilizing the Danny Platform (Sqilline Health, Sofia, Bulgaria). Treatment outcome was evaluated based on overall survival (OS), progression-free survival (PFS), objective response rate (ORR), and clinical benefit rate (CBR). Iterative proportional fitting was performed to improve comparability between the real-world and trial populations by accounting for differences in baseline characteristics. **Results**: Baseline characteristics were largely comparable among the ARCHES and our real-world cohort, with the most notable differences observed in prior systemic treatment exposure. A considerably higher ORR was reported for ARCHES (85.9%) in contrast to our cohort descriptive ORR (12.3%) derived from physician EHR notes, likely reflecting routine clinical documentation practices, whereas CBR values were comparable (89.6% in our cohort versus 95.9% in ARCHES). While median values were not calculable, estimated PFS and OS were consistent with those in ARCHES. **Conclusions**: Real-world outcomes from our Bulgarian cohort of patients with mHSPC are largely consistent with those observed among enzalutamide-treated participants in the ARCHES trial.

## Simple Summary

The ARCHES clinical trial demonstrated the clinical efficacy of combining androgen deprivation therapy, a well-established therapeutic option, with the next-generation drug enzalutamide for the treatment of metastatic prostate cancer. Nevertheless, the trial evaluated a limited number of patients compared to those receiving the combination in real-world clinical practice after its approval. Thus, it is essential to evaluate data from real-world patients and determine if it supports the findings of the ARCHES trial. Here, we present real-world evidence from Bulgarian patients with prostate cancer who received the combination. Our results regarding survival and the response of patients to treatment align closely with the findings of the ARCHES trial, supporting the use of enzalutamide in combination with androgen deprivation therapy for the treatment of metastatic prostate cancer.

## 1. Introduction

Prostate cancer accounts for up to 29% of newly diagnosed cancer cases in men, ranking as the second most common malignancy in men [1,2]. With over 1.2 million new diagnoses each year and more than 350,000 deaths, it is among the leading causes of cancer-associated death in men [3,4]. Incidence is related to life expectancy, as disease risk increases considerably with age, that is, more than 85% of patients are over 60 years of age at the time of diagnosis [5]. The strong relationship between prostate cancer risk and age underpins the greater incidence in developed regions with longer life expectancies [2]. Notably, prostate cancer is the malignancy with the highest incidence among individuals with a family history of cancer (~9% of diagnosed patients have such a history) [6,7]. A patient is defined as having familial prostate cancer if there are three or more affected relatives, of whom at least two develop prostate cancer before the age of 55 [8].

Most new prostate cancer diagnoses (~70%) are of organ-confined tumors, while locoregional and distant metastases are observed in 14% and 8% of cases, respectively [1]. Patients diagnosed with localized disease have a 10-year life expectancy as high as 99%, while those with distant metastases (late-stage disease) have a considerably poorer prognosis, with a 5-year survival rate of ~30% [1]. Among cases of metastatic disease, 55% are observed at initial diagnosis, and the rest as recurrence after early-stage disease and treatment (~45% of cases) [9,10]. Exploiting this dependency, androgen depletion therapy (ADT) is a mainstay in prostate cancer treatment [5]. Over half of patients with metastatic prostate cancer who receive ADT as initial treatment, experience a favorable response, and, based on the exhibited sensitivity, their disease is referred to as hormone-sensitive prostate cancer (HSPC). ADT is continued along with subsequent therapy even after sensitivity fades [11].

Over the past two decades, a number of treatment options have been developed for patients who progress on ADT [5]. These are usually given in parallel to continued ADT and include next-generation androgen receptor pathway inhibitors (ARPIs) [12]. Following the substantial survival benefits reported in the landmark ARCHES trial, enzalutamide received regulatory approval and, since 2019, has become a standard of care for metastatic HSPC (mHSPC), given in combination with ADT [13,14,15]. Longer times to prostate-specific antigen (PSA) progression, subsequent antineoplastic therapy, castration resistance, and the first symptomatic skeletal event were reported for the combination [16]. The survival benefit of enzalutamide has since been demonstrated for non-metastatic disease as well [17].

In light of the widespread use of enzalutamide for the treatment of mHSPC, collecting real-world insight is essential for corroborating clinical trial results, optimizing treatment, and improving patient outcomes. At present, there is a limited body of real-world evidence on enzalutamide plus ADT in mHSPC [18,19,20,21]. Herein, we retrospectively analyzed data from 48 hospitals across Bulgaria to assess clinical outcomes of enzalutamide plus ADT. Comparison of our results with those of ARCHES demonstrated that real-world efficacy largely aligns with that reported in the pivotal trial.

## 2. Materials and Methods

### 2.1. Data Source

Real-world patient data were retrospectively extracted from the electronic health records (EHRs) of patients from 48 university, multispecialty, and oncology hospitals across Bulgaria. Large language model (LLM) algorithms were employed for data extraction from structured and unstructured text. Analysis was performed using the Danny Platform (Sqilline Health, Sofia, Bulgaria; https://sqilline.com/), which integrates large amounts of real-world data from EHRs using embedded machine learning and LLM algorithms, which enable information extraction from free text in different languages. Further manual checks and/or annotation for consistency, errors, and missing values were performed, wherever applicable.

### 2.2. Endpoints

Treatment outcome was assessed based on overall survival (OS), progression-free survival (PFS), objective response rate (ORR), and clinical benefit rate (CBR). The OS rate was defined as the proportion of patients still alive a given period of time after treatment initiation. The PFS rate was defined as the proportion of patients living with disease for a given period after initiation, without experiencing disease progression. ORR was defined as the proportion of patients achieving a complete or partial response to the treatment administered for a given period. CBR was defined as the proportion of patients experiencing a complete response, partial response, or stable disease after receiving treatment for a given period of time. Mortality status was obtained from the unified system for civil registration and administrative services of the population (USCRASP), with mortality information available up to 31 December 2025.

### 2.3. Survival Analyses

PFS and OS were analyzed using the Kaplan–Meier estimator, with the confidence intervals calculated via Greenwood’s method. To account for differences in baseline characteristics between the real-world and trial cohorts, which may bias their subsequent interpretation, iterative proportional fitting (IPF) was performed. Thus, a set of baseline characteristics was selected prior to survival analysis, and weights were derived to adjust underlying distributions so that these would match a target distribution (i.e., that of the clinical trial). The characteristics collectively considered for IPF were age, ECOG performance status, total Gleason score at initial diagnosis, localization of confirmed metastases at screening, the presence of distant metastasis at initial diagnosis, disease volume, prior local therapy, and previous ADT use. Median follow-up was estimated using the reverse Kaplan–Meier method.

### 2.4. Statistical Analysis

Categorical baseline characteristics were compared using Pearson’s chi-square or Fisher’s exact test, as appropriate. Statistical significance was defined at 0.05. The 95% confidence intervals for ORR and CBR were calculated using the Wilson score method for binomial variables.

## 3. Results

### 3.1. Patient Selection

A total of 425 patients with mHSPC received enzalutamide plus ADT as a first-line treatment regimen between 1 January 2022 and 31 December 2025 across the hospitals included in this study. Based on the ARCHES inclusion criteria, patients were divided into an ARCHES-like treatment group (*N* = 249, 58.6%) and a non-eligible group (*N* = 176, 41.4%). The ARCHES-like treatment group included patients with mHSPC who received enzalutamide plus ADT as first-line treatment, had an ECOG performance status of 0 or 1, and had evidence of metastatic disease beyond the regional pelvic lymph nodes based on the clinical and radiological documentation in the EHRs (Figure S1) [13]. The ARCHES-like treatment group provided a substantial real-world cohort that aligned with the baseline population of the trial. Patients in the non-eligible group did not satisfy the main ARCHES eligibility criteria in terms of treatment history, metastatic disease characteristics, or other baseline features. Patients with prior exposure to second-generation ARPIs were excluded, in accordance with ARCHES eligibility criteria [13].

### 3.2. Prior Therapy

Prior systemic therapies within the ARCHES-like treatment group included adjuvant ADT (*N* = 104, 41.7%) with leuprorelin (*N* = 86, 34.5%) or goserelin (*N* = 18, 7.2%) and bicalutamide plus ADT (*N* = 5, 2.0%) (Table 1). No subsequent therapies were recorded among patients in this group during the available follow-up period. Patients with documented progression generally remained on enzalutamide, which was not considered subsequent therapy. Within the non-eligible group, adjuvant ADT with leuprorelin (*N* = 71, 40.3%) or goserelin (*N* = 17, 9.66%) was the most common prior systemic therapy (*N* = 88, 50%), followed by bicalutamide plus ADT (*N* = 14, 7.95%). Subsequent systemic therapy in the non-eligible group was recorded for 29 out of the 176 patients (*N* = 16.4%), with docetaxel plus leuprorelin (*N* = 16, 22.5%), abiraterone plus olaparib (*N* = 16, 22.5%), and abiraterone plus leuprorelin (*N* = 12, 16.9%) being most common. Docetaxel plus leuprorelin was also the most common subsequent systemic therapy received directly after enzalutamide plus ADT (*N* = 11, 6.3%).

Hereafter, we set out to compare baseline characteristics and clinical outcomes among our ARCHES-like treatment group and the actual trial cohort.

### 3.3. Baseline Characteristics

Median age in the real-world cohort was 74.0 years versus 70.0 years in the ARCHES trial, suggestive of an older patient population in real clinical practice (Table 2). The age group distribution further corroborated this difference, as a considerably larger proportion of patients fell within the ≥75 years group in the ARCHES-like treatment group when compared to the actual trial (47.4% versus 29.6%, respectively). In addition, patients under the age of 65 accounted for a smaller proportion in our real-world cohort (13.6% versus 25.8%). As per inclusion criteria, patients in either cohort had an ECOG status of 0–1. However, a pronounced difference was observed for the ECOG distribution, as most patients (*N* = 179, 71.9%) from the ARCHES-like treatment group were ECOG 1, while trial participants were mostly ECOG 0 (*N* = 448, 78.2%) (Table 2). Such a pattern suggests that those treated in the real-world setting are more likely to begin therapy with mild functional impairment. Meanwhile, Gleason scores were consistent between the two populations, as 67.6% and 69.3% of patients had a score ≥8 in the real-world and trial cohorts, respectively. Disease aggressiveness is thus generally aligned between the two cohorts.

The overall rate of confirmed metastases at screening was similar, albeit slightly lower (90.7% versus 93.4%) in the real-world cohort (Table 2). In a small proportion of patients, disease was classified as ‘not metastatic’ at screening (7.6% versus 5.9%) or unknown (1.6% versus 0.7%), without a statistically significant difference (*p* = 0.302). Modest differences in metastatic localization were noted among patients with confined metastases, with a higher proportion of bone-only disease (55.3% versus 46.7%) and soft-tissue-only disease (12.8% versus 8.9%) in the real-world cohort. Meanwhile, combined bone and soft tissue metastases were less frequent (31.9% versus 37.8%) among real-world patients, albeit without statistical significance (*p* = 0.059) (Table 2). Differences were also present in distant metastases at initial diagnosis but did not reach statistical significance (*p* = 0.088). In the real-world cohort, M1 disease was more common (77.5% versus 70.2%), while M0 (11.6% versus 14.5%) and MX/unknown (10.8% versus 15.3%) were somewhat less frequent than in the ARCHES trial population. High-volume disease (defined per CHAARTED criteria as the presence of visceral metastases or, in their absence, ≥4 bone lesions, including at least one beyond the vertebral column or pelvis) was slightly less common among the real-world cohort (54.6% versus 61.7%), with an accordingly higher proportion of low-volume disease noted (45.4% versus 38.3%), yet without statistical significance (*p* = 0.058).

Prior local treatment was less common in our real-world cohort when compared to the ARCHES trial, as radical prostatectomy had been performed in 4.4% of patients as opposed to 12.5% in the trial (Table 2). Radiation therapy was reported in 10.0% and 12.7% of real-world and trial patients, respectively. The most pronounced differences were noted in prior systemic treatment exposure. None of the patients in the real-world cohort had received prior docetaxel, as opposed to 17.9% in the ARCHES trial (of whom 2.4% with 1–5 cycles and 15.5% with 6 cycles). Likewise, previous ADT exposure was much less common among real-world patients, as 58.2% had not received any, compared to only 6.8% in the ARCHES cohort. Among those with prior ADT exposure, limited prior ADT use (≤3 months) was more common in the real-world cohort than the trial cohort (86.5% versus 77.4%). Accordingly, longer prior ADT exposure (>3 months) was less frequent (13.5% versus 22.6%). The median duration of prior ADT was shorter in the real-world cohort when compared to ARCHES (0.9 versus 1.6 months, respectively). Prior antiandrogen use was markedly less frequent among real-world patients (2.0% versus 35.8%). Another large difference was noted in median PSA at enzalutamide initiation, with 48.5 versus 5.4 ng/mL in the real-world and trial cohorts, respectively.

### 3.4. Survival Analysis

We employed IPF to balance the above-described baseline characteristics, aligning them with those reported in the ARCHES trial in order to perform comparative survival analysis. Standardized mean differences before and after weighting for all balancing covariates are provided in Supplementary Table S1. The final normalized weights had a mean of 1.00, a median of 0.71 (IQR 0.56–1.70), and ranged from 0.16 to 2.69, corresponding to an effective sample size of approximately 180 patients from the original cohort of 249. Some residual imbalance remained, particularly for ECOG status and prior ADT duration. Prior docetaxel exposure, prior antiandrogen treatment, and baseline PSA were not weighted due to limited common support and remained substantially imbalanced. Within the real-world cohort, the descriptive ORR was 12.3% (95% CI 8.4–17.5), which was considerably lower than that reported in the trial (85.9% [95% CI 79.9–90.3]) (Table 3). We attribute this difference, at least in part, to real-world documentation practices, whereby patients are often recorded as having stable disease rather than complete or partial responses, which contributes to a lower descriptive ORR. In line with this notion, the difference in CBR was much smaller, at 89.6% (95% CI 84.7–93.1) versus 95.9% (95% CI 91.7–98.0) in the real-world and ARCHES cohorts, respectively (*p* = 0.022).

**Figure 1:**
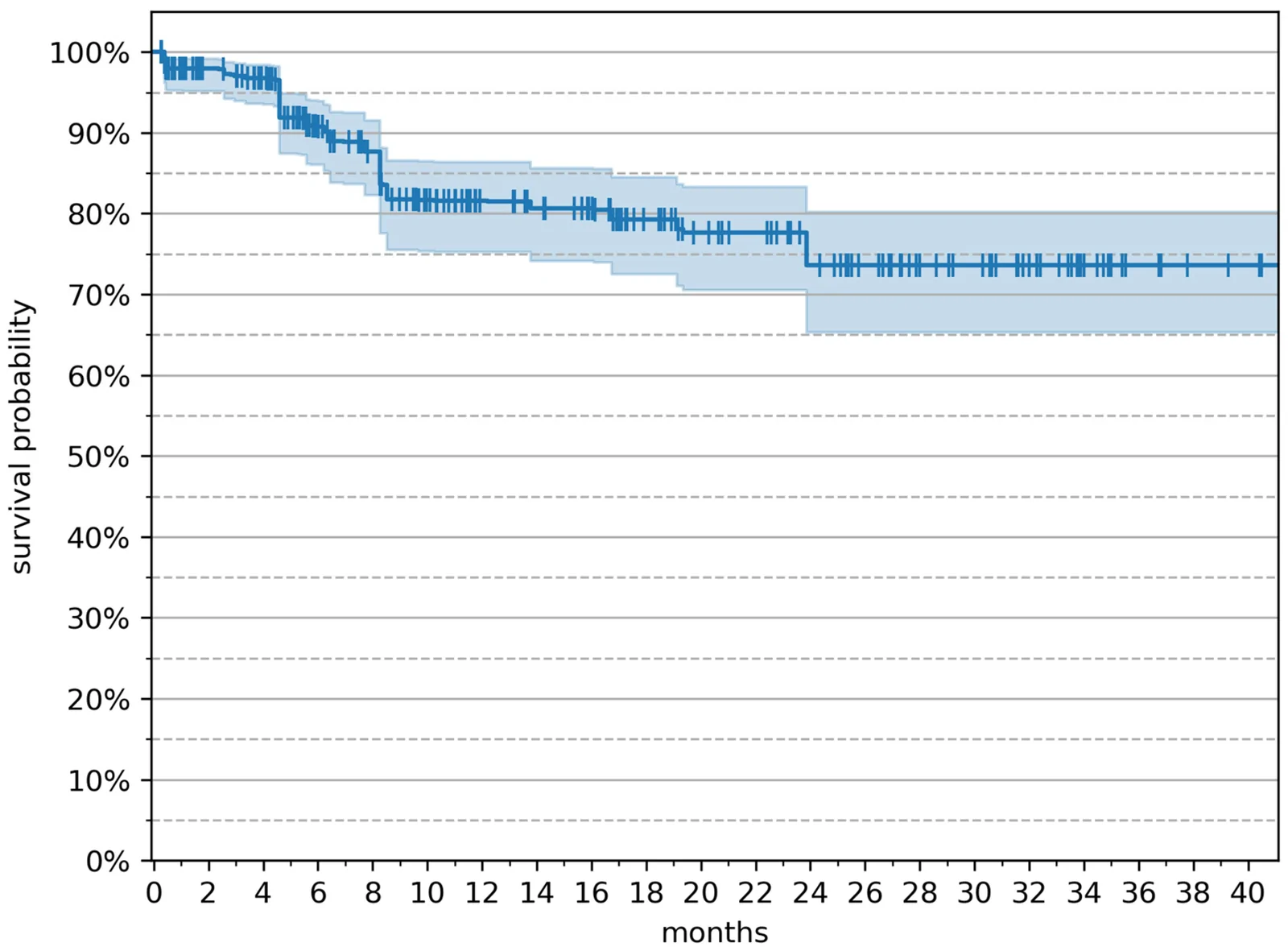
Kaplan–Meier estimate of adjusted PFS in the real-world cohort.

Median follow-up in the RWE cohort was 16.1 months (95% CI 12.7–18.0), during which median PFS could not be calculated in either cohort. IPF-adjusted PFS estimates in the real-world cohort were broadly similar to the published ARCHES estimates during the period with sufficient numbers at risk (Table 4). Nevertheless, slightly higher early PFS estimates (from 6 to 12 months) were noted in the trial, at 91% vs. ~94% at 6 months, 82% versus 88% at 9 months, and 82 versus 83% at 12 months. From 15 to 21 months, this slight difference shifted in favor of the real-world cohort, with 81% versus ~79% at 15 months, 79% versus ~75% at 18 months, and 78% versus ~73% at 21 months. PFS remained slightly higher in the real-world cohort at both 24 and 27 months (74% versus ~73% for both) (Figure 1). These later comparisons should be interpreted with caution, as very few patients remained at risk in the ARCHES arm.

**Figure 2:**
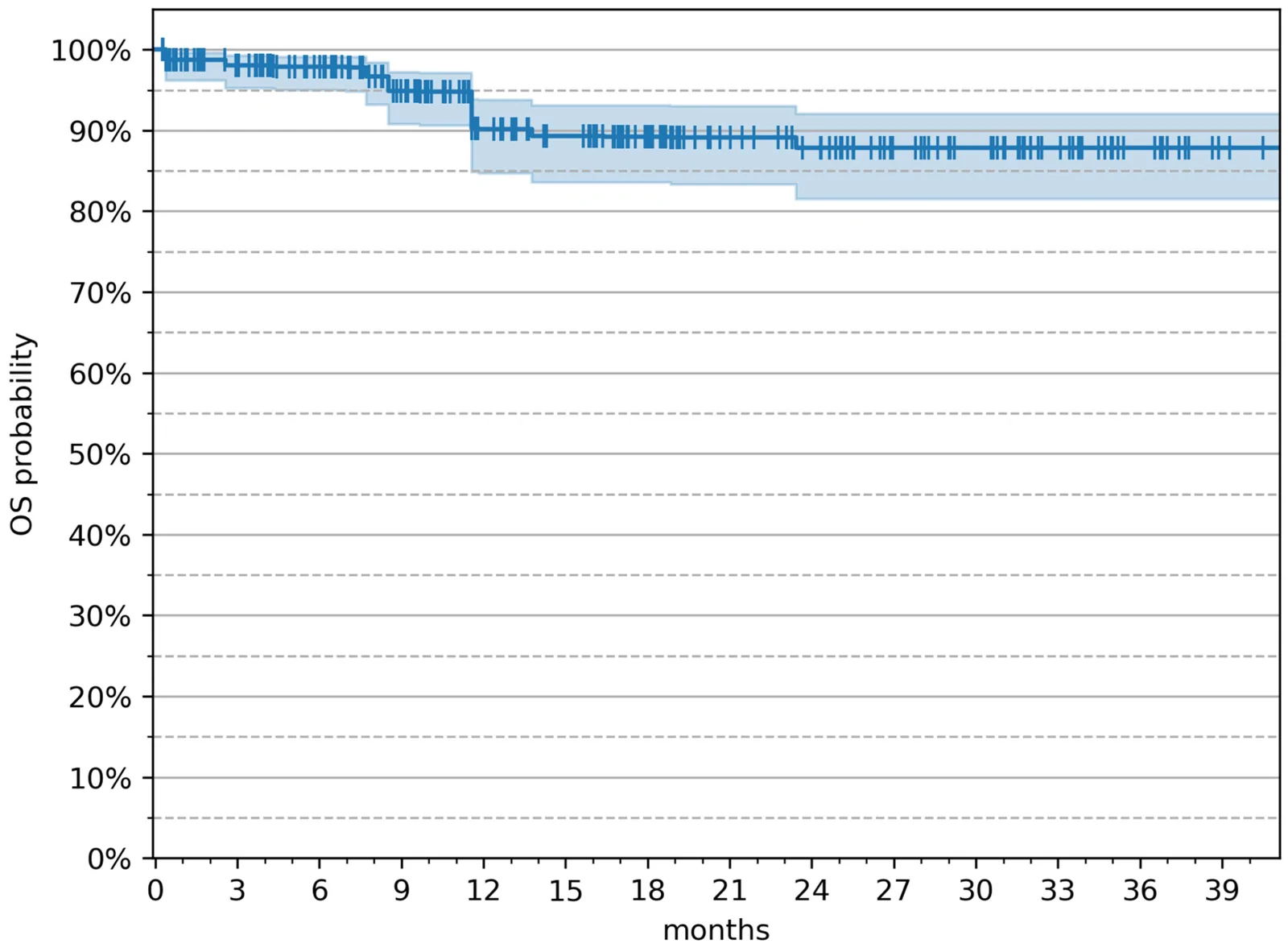
Kaplan–Meier estimate of adjusted OS in the real-world cohort.

Median OS was also not calculable in our cohort. A markedly lower number of OS events was recorded in the real-world versus trial cohort (16 [6.4%] versus 191 [33.3%]). Despite this difference, IPF-adjusted OS was highly similar during earlier follow-up. Estimates were slightly lower in the real-world cohort during the first year (98% versus ~99% at 6 and 90% versus 96% at 12 months) (Table 4). OS estimates were also similar at 18 and 24 months, with 89% versus ~91% and 88% versus 86%, respectively. At 30 and 36 months, OS remained stable at 88%, while that in the ARCHES cohort declined to 82% and 78%, respectively (Figure 2). In light of the substantial decrease in patients at risk within the real-world cohort, comparisons at these later timepoints should be interpreted with caution.

## 4. Discussion

The past two decades have seen the introduction of novel treatment options for patients with prostate cancer, with the second-generation ARPI enzalutamide emerging as a standard-of-care treatment in mHSPC. While the pivotal ARCHES and ENZAMET trials strongly support its clinical benefit, corroboration of trial findings via real-world studies of much larger and diverse patient cohorts is warranted [13,15]. In line with this notion, the current work provides valuable real-world insights into the efficacy of enzalutamide plus ADT for the treatment of mHSPC, allowing for a descriptive comparison with published ARCHES trial results.

To reduce observed differences in baseline characteristics between the trial and the real-world populations, IPF was performed. Nevertheless, certain differences in patient baseline characteristics were noted. The real-world cohort had a higher median age and a larger proportion of patients within the ≥75 years age group. This is in line with the median age reported for the ARON-3 real-world study of enzalutamide plus ADT in mHSPC, which was 72.0 years and thus also higher than in ARCHES [18]. A larger proportion of patients in our real-world cohort had ECOG 1 performance status (71.9%), as opposed to the predominant ECOG 0 status among ARCHES participants (78.2%). The ARON-3 cohort also included a higher proportion of patients with ECOG 1 status compared to ARCHES (38% versus 22%), albeit considerably lower than in our cohort [18]. These observations are in line with the general notion that patients in real-world practice are older and have a poorer performance status compared to trial participants [22]. Gleason scores were slightly higher in our cohort when compared to ARCHES participants (67.6% versus 69.3%), consistent with an older patient population and poorer performance status. Nevertheless, disease aggressiveness was consistent between the trial and real-world cohorts. High-volume disease (as per CHAARTED criteria) was less common in both our real-world cohort (54.6%) and the ARON-3 real-world study (46%) when compared to ARCHES (61.7%) [18,23]. This may reflect a trial selection bias, as patients with measurable or extensive disease are easier to evaluate, and events tend to occur sooner in such a cohort. A lower proportion of high-volume disease could also entail a better prognosis. It should be noted that the lower rates of high-volume disease may stem from advances in diagnosis, where metastases can be detected at an earlier stage, before reaching high-volume disease. Low-volume disease was more common both in our cohort (45.4%) and ARON-3 (54%), when compared to ARCHES (38.3%). A similar trend was observed in both real-world cohorts compared to the trial with regard to bone-only disease, reported in 55.3% of patients in our cohort versus 46.7% in ARCHES and 63% in ARON-3 [13,16,18]. While bone-only disease has been associated with a more favorable prognosis when compared to disease with visceral metastases, this classification does not imply a lower metastatic burden, as extensive skeletal involvement may be observed [16]. Soft-tissue-only disease was more frequent in our cohort (12.8%) and ARON-3 (19%), when compared to 8.9% in ARCHES [16,18]. In both ARON-3 and the ARCHES post hoc analysis, it was concluded that patients with soft-tissue-only disease experienced better treatment outcomes when compared to those with bone-only disease, as determined by changes in PSA levels, time on treatment, and overall survival [16,18]. Patients with visceral disease had the least favorable prognosis among the three metastatic patterns. Interestingly, combined bone and soft tissue metastases were less frequent within our real-world cohort (31.9%) when compared to ARCHES (37.8%). As no data on combined bone and soft tissue metastases were provided in the ARON-3 study, the current work provides a valuable patient subgroup in terms of baseline metastatic patterns, which can be directly compared to ARCHES data.

In terms of prior local treatment, radical prostatectomy was considerably less common in our real-world cohort (4.4%) compared to ARCHES (12.5%). Meanwhile, this proportion was nearly five- and two-fold greater (~25%) in the ARON-3 cohort, respectively. Radiotherapy rates were comparable between ARCHES (12.7%), our study (10.0%), and ARON-3 (~11%) [13,18]. A notable difference between our real-world cohort and ARCHES is the rate of prior docetaxel use, where 17.9% of the trial cohort had received docetaxel versus none in the real-world cohort. This may complicate baseline comparisons to patients who have not received prior docetaxel. In the ARON-3 study, patients with prior docetaxel use experienced shorter median times on enzalutamide [18]. However, it should be noted that in the ARCHES trial there was no significant interaction between the efficacy of enzalutamide plus ADT and prior chemotherapy, that is, progression-free survival was unaffected by prior docetaxel treatment [13]. Thus, while prior docetaxel use may be prognostic, as it has been historically administered to patients with high-volume, aggressive metastatic disease, it is not a predictive factor in relation to the clinical benefit of enzalutamide [24,25]. There was a substantial difference in prior antiandrogen use between our real-world cohort (2.0%) and the ARCHES cohort (35.8%). While this was a considerable difference, a post hoc analysis of ARCHES demonstrated that prior antiandrogen use was not associated with differences in PFS and secondary clinical outcomes [26]. Taken together, the most pronounced differences in our comparison of real-world versus ARCHES data were observed in prior systemic treatment exposure, with markedly less prior use of ADT, antiandrogens, and docetaxel recorded among real-world patients.

Another noteworthy difference was observed in median PSA levels at enzalutamide initiation, which were several-fold higher in our cohort (48.5 ng/mL) compared to both ARCHES (5.4 ng/mL) and the ARON-3 cohort (18.3 ng/mL) [13,18]. This difference was consistent with the more limited prior ADT exposure in our real-world cohort. It should be noted that PSA values are influenced by the timing of measurement, documentation practices in the real world, as well as differences in baseline disease burden. More importantly, more than half of patients in our real-world cohort had not received prior ADT, compared to only 6.8% in the ARCHES trial, which is a likely contributor to the difference in PSA levels.

In terms of tumor response, there was a significant difference in our real-world descriptive ORR (12.3%) in contrast with the ARCHES study ORR (85.9%). However, this is attributed to real-world documentation practices where patients are often recorded as having stable disease instead of complete or partial responses. Challenges in achieving the same degree of standardization based on RECIST criteria in real-world clinical practice have been previously acknowledged [27]. In support of this notion, the clinical benefit rates were much closer, at 89.6% versus 95.9% for real-world and trial data. ARON-3 reported an ORR and CBR of 53% and 86%, respectively [18]. Median PFS and OS data were not available for our real-world cohort. Nevertheless, PFS estimates were consistently comparable to trial data, with the exception of later months (after month 24), where very few patients had remained at risk in the ARCHES arm. With regard to OS, there was a markedly lower number of recorded OS events in the real-world cohort. Nevertheless, IPF-adapted OS was broadly similar between the real-world and trial cohorts throughout follow-up. Of note, OS in the real-world cohort remained stable at later timepoints, while a decline was observed in the ARCHES arm, which warrants caution when interpreting later comparisons, especially considering the fact that patients at risk in our real-world cohort decreased substantially after the 30th month. Taken together, tumor response and survival data from our real-world cohort were largely consistent with the ARCHES findings, supporting the use of enzalutamide plus ADT in mHSPC. This is in agreement with the conclusions of the ARON-3 real-world study [18].

While the current work provides a valuable contribution to the limited body of evidence on the real-world efficacy of enzalutamide plus ADT in mHSPC, several limitations should be noted. The median time on treatment (ToT) is a valuable addition to PFS data, which takes into consideration patients who had to stop treatment [27]. Assessing ToT would further corroborate the clinical benefit observed in our real-world cohort. In addition, PSA90 and PSA0.2 have been described as useful on-treatment predictors with regard to PFS, OS, and ToT based on ARON-3 and ARCHES post hoc analyses [18,28]. The evaluation of PSA response patterns thus represents another potential point of improvement. Time to castration resistance represents another potentially informative endpoint for future real-world analyses. In addition, our real-world results do not address the safety of enzalutamide plus ADT, such as the rate of severe adverse events. In this regard, patient comorbidities represent another relevant aspect in real-world clinical practice and warrant further consideration. Further characterization of the non-ARCHES-eligible population may provide additional real-world insights and should be considered in future research. The current work has limitations inherent to its retrospective real-world design, which may introduce selection and information biases, in addition to incomplete clinical documentation and limited capture of treatment received outside participating institutions. Residual imbalance in selected baseline characteristics remained after weighting and may contribute to residual confounding. Published ARCHES survival estimates used for external comparison were based on aggregate trial-level data and are therefore presented descriptively rather than as a formal patient-level survival comparison.

## 5. Conclusions

In conclusion, the results of the current work suggest that real-world efficacy data for enzalutamide plus ADT in mHSPC were broadly consistent with those reported in the pivotal ARCHES trial. While certain differences in baseline characteristics, such as older age, poorer performance status, and baseline treatment exposure were noted, descriptive IPF-adjusted PFS and OS estimates in the real-world cohort were generally similar to published ARCHES estimates during earlier follow-up. Although the discrepancy in CBR between the real-world and trial cohorts was smaller than that observed for ORR, the real-world CBR was still lower in contrast to the ARCHES study (95.9% versus 89.6%, respectively). Taken together, the current findings show that real-world outcomes for enzalutamide plus ADT align closely with the data from the pivotal trial.

## Figures and Tables

**Table 1:** Prior systemic therapy in the ARCHES-like treatment group.

Regimen	Number of Regimens	Number of Regimens, %
Adjuvant ADT	104	41.7%
Leuprorelin	86	34.5%
Goserelin	18	7.20%
Bicalutamide + ADT	5	2.00%

**Table 2:** Baseline characteristics in the real-world and ARCHES trial cohorts.

Characteristic	Values	RWE	Clinical Trial % (*N* = 574)
		% (*N* = 249)	
Age (at start)		years	
Median		74.0	70.0
Mean ± SD		72.9 ± 7.2	-
Q1–Q3		68.0–78.0	-
Min–Max		54–91	46–92
Age group		% (*N* = 249)	% (*N* = 574)
<65		13.6 (34)	25.8 (148)
65–74		39.0 (97)	44.6 (256)
≥75		47.4 (118)	29.6 (170)
ECOG		% (*N* = 249)	% (*N* = 573)
0		28.1 (70)	78.2 (448)
1		71.9 (179)	21.8 (125)
Total Gleason score at initial diagnosis		% (*N* = 213)	% (*N* = 557)
<8		32.4 (69)	30.7 (171)
≥8		67.6 (144)	69.3 (386)
Confirmed metastases at screening		% (*N* = 249)	% (*N* = 574)
Yes		90.7 (226)	93.4 (536)
No		7.6 (19)	5.9 (34)
Unknown		1.6 (4)	0.7 (4)
Localization of confirmed metastases at screening		% (*N* = 226)	% (*N* = 536)
Bone only		55.3 (125)	46.7 (268)
Soft tissue only		12.8 (29)	8.9 (51)
Bone and soft tissue		31.9 (72)	37.8 (217)
Distant metastasis at initial diagnosis		% (*N* = 249)	% (*N* = 573)
M1		77.5 (193)	70.2 (402)
M0		11.6 (29)	14.5 (83)
MX/unknown		10.8 (27)	15.3 (88)
Disease volume		% (*N* = 249)	% (*N* = 574)
High		54.6 (136)	61.7 (354)
Low		45.4 (113)	38.3 (220)
Prior local therapy		% (*N* = 249)	% (*N* = 574)
Radical prostatectomy		4.4 (11)	12.5 (72)
Radiation therapy		10.0 (25)	12.7 (73)
No. of cycles of prior docetaxel chemotherapy		% (*N* = 249)	% (*N* = 574)
0		100 (249)	82.1 (471)
1–5		0 (0)	2.4 (14)
6		0 (0)	15.5 (89)
Previous use of ADT, months		% (*N* = 249)	% (*N* = 574)
None		58.2 (145)	6.8 (39)
≤3		36.1 (90)	72.1 (414)
>3		5.6 (14)	21.1 (121)
Duration of prior ADT among exposed patients, months		% (*N* = 104)	% (*N* = 535)
≤3		86.5 (90)	77.4 (414)
>3		13.5 (14)	22.6 (121)
Median duration of prior ADT, months (range)		0.9 (0.22–5.94)	1.6 (0.03–55.3)
Previous use of antiandrogen, % (N)		2.0 (5)	35.8 (205)
Median PSA, ng/mL (range)		48.5 (0–9 578.0)	5.4 (0–4 823.5)

**Table 3:** Tumor responses in the real-world and ARCHES trial cohorts.

Best Response	*N* = 249	RWE with Response % (*N* = 203)	*N* = 177	Clinical Trial with Response % (*N* = 171)
Complete Response	4	2.0	65	38.0
Partial Response	21	10.3	82	47.9
Stable Disease	157	77.3	17	10.0
Progression	21	10.3	7	4.1
Not evaluated	46	-	6	-
	***N***	**% (*N* = 203)**	***N***	**Clinical Trial** **% (*N* = 171)**
ORR (Objective Response Rate)	25	12.3 (8.4–17.5)	147	85.9 (79.9–90.3)
CBR (Clinical Benefit Rate)	182	89.6 (84.7–93.1)	164	95.9 (91.7–98.0)

**Table 4:** Overall and progression-free survival in the real-world and ARCHES cohort.

		RWE		ARCHES	
Survival		Patients at Risk	*N* = 249	Patients at Risk	*N* = 574
**PFS**					
	number of events		45 (18.0%)		89 (15.5%)
	Median		NC (NC–NC)		NC (NC–NC)
	at 3 mo.	211	97% (94–99)	516	~96%
	at 6 mo.	162	91% (86–94)	493	~94%
	at 9 mo.	139	82% (75–87)	370	~88%
	at 12 mo.	111	82% (75–86)	256	83%
	at 15 mo.	103	81% (74–86)	144	~79%
	at 18 mo.	81	79% (72–85)	62	~75%
	at 21 mo.	67	78% (71–83)	23	~73%
	at 24 mo.	58	74% (65–80)	4	~73%
	at 27 mo.	43	74% (65–80)	1	~73%
**OS**					
	number of events		16 (6.4%)		191 (33.3%)
	Median		NC (NC–NC)		NC (83.1–NC)
	at 6 mo.	195	98% (95–99)	559	~99%
	at 12 mo.	136	90% (85–94)	535	96%
	at 18 mo.	101	89% (84–93)	498	~91%
	at 24 mo.	73	88% (82–92)	457	86%
	at 30 mo.	46	88% (82–92)	427	~82%
	at 36 mo.	19	88% (82–92)	396	78%

Abbreviations: NC, not calculable; “~” indicates an approximate value derived from the published ARCHES Kaplan–Meier figure.

## Data Availability

Data supporting reported results can be obtained from the corresponding author upon reasonable request.

## Supplementary Materials

The following supporting information can be downloaded at: https://www.mdpi.com/article/10.3390/cancers18183007/s1, Table S1: Baseline covariate balance before and after iterative proportional fitting (IPF); Figure S1: Flow chart for patients with mHSPC treated with enzalutamide plus ADT.

## Abbreviations

The following abbreviations are used in this manuscript:

ADT	Androgen Deprivation Therapy
CBR	Clinical Benefit Rate
CI	Confidence Interval
EHR	Electronic Health Record
IPF	Iterative Proportional Fitting
mHSPC	Metastatic Hormone-Sensitive Prostate Cancer
ORR	Objective Response Rate
OS	Overall Survival
PFS	Progression-free Survival
PSA	Prostate-specific Antigen
