# Supplementary material for: Enzalutamide Plus ADT for the Treatment of mHSPC: Real-World Evidence from a Bulgarian Cohort
Source: Cancers (Basel). 2026 Sep 16;18(18):3007. doi: 10.3390/cancers18183007 (PMC13604666; doi:10.3390/cancers18183007)
Supplement: Supplementary file 1 [file cancers-18-03007-s001.zip › cancers-4515322-supplementary.pdf]

Supplementary Table S1. Baseline covariate balance before and after iterative proportional fitting (IPF).

Baseline covariate	RWE unweighted	RWE weighted	ARCHES	Pre-IPF ∣SMD∣	Post-IPF ∣SMD∣
Age <65 years	13.7%	22.5%	25.8%	0.308	0.077
Age 65 – 74 years	39.0%	43.5%	44.6%	0.114	0.022
Age ≥ 75 years	47.4%	34.0%	29.6%	0.372	0.095
ECOG 0	28.1%	61.2%	78.2%	1.161	0.376
ECOG 1	71.9%	38.8%	21.8%	1.161	0.376
Gleason <8	32.4%	31.2%	30.7%	0.037	0.011
Gleason ≥8	67.6%	68.8%	69.3%	0.037	0.011
Confirmed metastases: Yes	90.7%	92.1%	93.4%	0.100	0.050
Confirmed metastases: No	7.6%	6.5%	5.9%	0.068	0.025
Confirmed metastases: Unknown	1.6%	1.4%	0.7%	0.085	0.068
Localization: Bone only	55.3%	51.5%	50.0%	0.106	0.030
Localization: Soft tissue only	12.8%	10.2%	9.5%	0.105	0.023
Localization: Bone + soft tissue	31.9%	38.3%	40.5%	0.180	0.045
Distant metastasis: M1	77.5%	72.8%	70.2%	0.167	0.058
Distant metastasis: M0	11.6%	14.1%	14.5%	0.084	0.011
Distant metastasis: MX/unknown	10.8%	13.1%	15.3%	0.134	0.061
Disease volume: High	54.6%	58.9%	61.7%	0.144	0.057
Disease volume: Low	45.4%	41.1%	38.3%	0.144	0.057
Prior local therapy: Radical prostatectomy	4.4%	8.5%	12.5%	0.295	0.133
Prior local therapy: Radiation therapy	10.0%	12.3%	12.7%	0.084	0.014
Prior local therapy: None	85.5%	79.3%	74.7%	0.273	0.108

Prior ADT: None	58.2%	18.5%	6.8%	1.313	0.358
Prior ADT: ≤3 months	36.1%	70.5%	72.1%	0.775	0.035
Prior ADT: >3 months	5.6%	11.0%	21.1%	0.468	0.278
Prior docetaxel: No	100.0%	100.0%	82.1%	0.660	0.660
Prior docetaxel: Yes	0.0%	0.0%	17.9%	0.660	0.660
Prior antiandrogen: No	98.0%	98.0%	64.2%	0.957	0.957
Prior antiandrogen: Yes	2.0%	2.0%	35.8%	0.957	0.957
Baseline PSA, median (range), ng/mL	48.5 (0 – 9 578.0)	42.1 (0 – 9 120.0)	5.4 (0 – 4 823.5)	–	–

Abbreviations: ADT, androgen deprivation therapy; ECOG, Eastern Cooperative Oncology Group; IPF, iterative proportional fitting; PSA, prostate-specific antigen; RWE, real-world evidence; SMD, standardized mean difference.

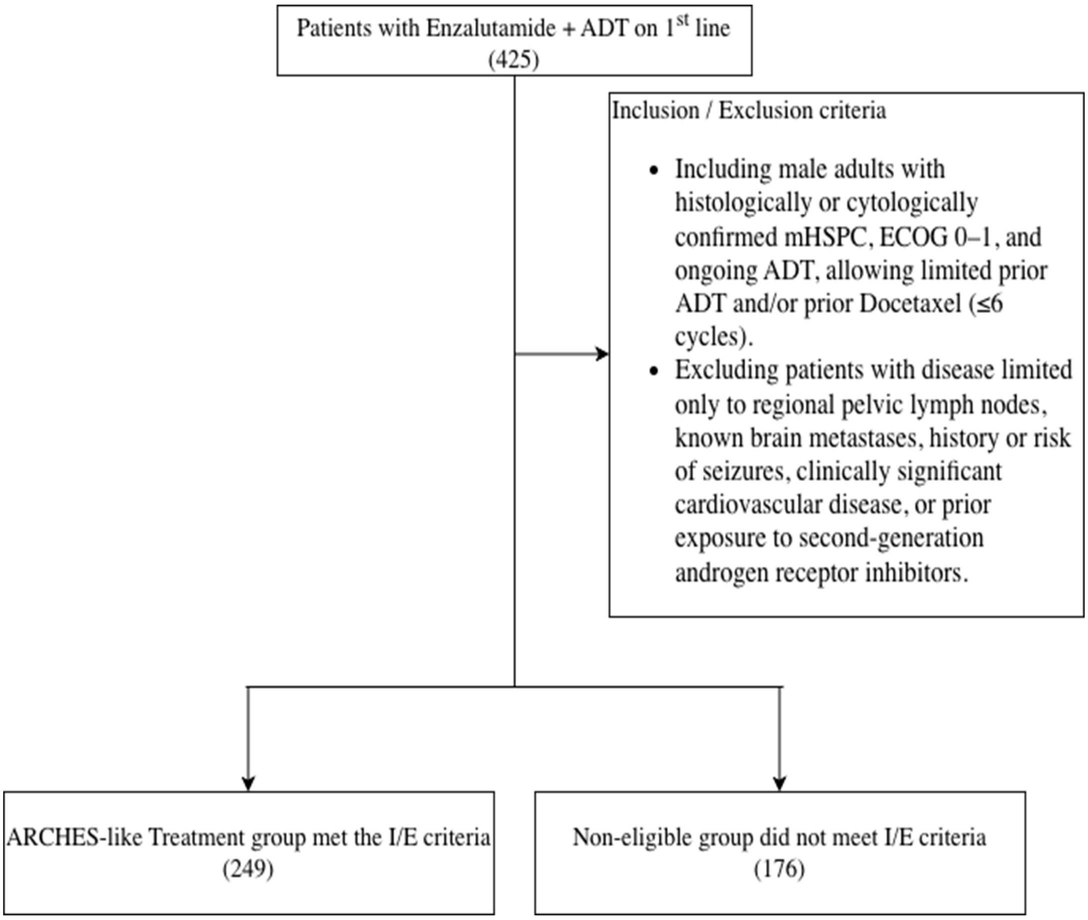

Figure S1. Flow chart for patients with mHSPC treated with enzalutamide plus ADT.
